# Health Equity Leadership and Mentoring (HELM): A health equity-focused early-career development program to promote biomedical workforce diversity

**DOI:** 10.1017/cts.2025.10083

**Published:** 2025-06-26

**Authors:** Melanie Steiner, Susan A. Everson-Rose, April F. Mohanty, José E. Rodríguez, Molly B. Conroy, Michele L. Allen, Antonia Apolinário-Wilcoxon, Kolawole S. Okuyemi

**Affiliations:** 1 Department of Family Medicine, Indiana University School of Medicine, Indianapolis, IN, USA; 2 Department of Family and Preventive Medicine, University of Utah Spencer Fox Eccles School of Medicine, Salt Lake City, UT, USA; 3 Department of Medicine, University of Minnesota Medical School, Minneapolis, MN, USA; 4 Department of Internal Medicine, University of Utah Spencer Fox Eccles School of Medicine, Salt Lake City, UT, USA; 5 Department of Family Medicine and Community Health, University of Minnesota Medical School, Minneapolis, MN, USA; 6 Equity Strategies, LLC, Minneapolis, MN, USA

**Keywords:** Career development, retention, workforce diversity, culturally aware mentoring, health equity leadership

## Abstract

Intentionally enhancing and supporting the early careers of individuals from populations underrepresented in science and medicine (URSM) is essential to achieving health equity. The Health Equity Leadership and Mentoring (HELM) Program at the University of Minnesota and the University of Utah is designed to foster academic excellence and build leadership capacity of postdoctoral fellows, clinical fellows, and early-career faculty who identify as URSM and/or who are committed to careers in health equity research and clinical care. HELM models a culture of psychosocial safety to create a sense of belonging and uses evidence-based and culturally aware mentoring and career development strategies with the goal of retaining diverse faculty. HELM proved agile and adaptive during the Covid-19 pandemic and has been successful in states with and without legislation limiting diversity programs. Across the 2 institutions, the HELM program has supported over 200 trainees and early-career faculty through mid-2024. Among HELM participants who joined the program as faculty, 85%–95% have remained in their faculty positions.

## Introduction

Health disparities in the USA are a result of documented historical as well as contemporary political, social, and economic factors that pattern access to healthcare, opportunities for healthy lifestyles, and health outcomes. Solutions to persistent health inequities include the development of a more diverse biomedical research and clinical workforce [1–3]. Intentionally enhancing and supporting the early-career development of individuals from backgrounds historically underrepresented in science and medicine (URSM) is thus essential to achieving improved health research, healthcare, and health equity [4,5].

The National Institutes of Health (NIH) defines individuals from racial and ethnic minority groups and women among populations underrepresented in biomedical research [6]. Specifically, the NIH has documented a “striking loss” of women overall and of men from underrepresented backgrounds from faculty ranks across the biomedical research career spectrum [7]. The NIH has also reported a marked and persistently lower prevalence of NIH research funds awarded to investigators who are from racial and ethnic groups underrepresented in biomedical research [8]. In response, the NIH has invested in the science of mentoring to inform programs designed to positively impact the transition from training to career independence. For example, a required component of NIH National Center for Advancing Translational Sciences (NCATS)-funded Clinical and Translational Science Award (CTSA) programs is to implement programming that enhances the career development of postdoctoral fellows and junior faculty, including those who identify as URSM, to meet the NCATS strategic objectives of building a workforce that reflects the diversity of communities and identities in the USA [9–11]. Similarly, the American Medical Association, the Association of American Medical Colleges, and other medical societies are committed to addressing structural and institutional health inequities. They note that “[e]xcellence in patient care cannot exist until we have a physician workforce capable of caring for our patients and their needs holistically, and until the profession of medicine is accessible to all qualified individuals” [12].

The Health Equity Leadership and Mentoring (HELM) Program is an intramurally funded program with the mission to foster academic excellence and leadership of postdoctoral fellows and clinical fellows (“trainees” hereafter) and early-career faculty who identify as URSM and/or who have committed to careers in health equity research, education, or clinical care. HELM does not replace discipline-specific mentoring but expands the mentoring landscape to include a cohesive and safe space in which to broach and discuss cultural identity within the contexts of career development and health equity leadership. HELM leans on evidence-based mentoring strategies developed by the NIH-funded National Research Mentoring Network (NRMN) [13–16] and mentor training curricula disseminated by the Center for the Improvement of Mentored Experience in Research (CIMER) [10, 17–19]. Drawing on these best practices established for research mentoring, HELM’s novel contribution to the development and advancement of trainees and early-career faculty is its intentional cultivation of psychosocial safety and a sense of belonging, with the goal of retaining faculty and building diverse leadership capacity from within the institution.

HELM was designed and implemented at the University of Minnesota (UMN) in 2014 and adapted by the University of Utah (UU) in 2018. Across the 2 institutions, the HELM program has supported over 200 early-career scholars through mid-2024.

In this report, we provide a conceptual overview of HELM and its distinctive implementation at UMN and UU, describing participants, program curricula, mentor–mentee matching and preparation, challenges, and outcomes. We highlight the program’s culture of modeling open communication, belonging, and respect for differences and thereby creating a safe space for URSM trainees and early-career faculty, which serves as the foundation for HELM’s three domains: mentoring, health equity-focused career and leadership development, and networking.

## Program overview and objectives

URSM trainees and faculty face numerous unique challenges, including marginalization, cultural and social isolation, imposter phenomenon, lack of role models and mentors with similar lived experiences, and overt and covert forms of sexism, racism, and discrimination [20–24]. Bias in hiring and promotion decisions, limited access to mentorship and networking opportunities, disproportionate expectations to contribute to service activities that may not count in promotion decisions (the “minority tax”), and lack of representation in leadership impede career development and advancement [25–30]. URSM trainees and faculty also face discrimination in the form of lower salaries, inadequate resources, and lack of recognition for their contributions [31,32]. Collectively, these experiences can negatively impact job satisfaction and mental health [33–35]. One objective of HELM is to provide URSM trainees and faculty with the psychosocial safety to name, address, and overcome these challenges. The desired outcome is the retention of diverse and health equity-focused faculty who thrive in their careers and make impactful gains in ending health disparities.

HELM uses a culturally responsive model of mentoring and career development to offer training, resources, and opportunities for participating trainees and early-career faculty, called HELM Fellows [10,36,37]. The program provides focused one-on-one career mentoring with an established faculty member, access to nationally recognized scholars and leaders in health equity and workforce diversity, and monthly seminars on professional and leadership development and other topics relevant to challenges faced by HELM Fellows. HELM operates on a cohort model, welcoming a new cohort at the beginning of each academic year.

HELM was developed at UMN in 2014 by senior faculty in the Departments of Medicine (SER) and Family Medicine and Community Health (KO), both with leadership appointments in the Program in Health Disparities Research. One of the directors relocated to UU in 2018. At UMN, HELM is currently directed by one faculty, and administrative support is provided by the Program in Health Disparities Research staff. The first HELM cohort at UU started in 2018 as an adaptation of the UMN program. Established faculty from the Departments of Family and Preventive Medicine (KO) and Internal Medicine (MC) served as inaugural program co-directors; an early-career faculty and 2018 HELM alumna (AM) joined as the third co-director in 2020. Until 2024, the research director (MS) and staff in the Department of Family and Preventive Medicine provided program support. Currently, the administrative home of UU HELM is the office of the Associate Vice President for Health Sciences Workforce Excellence (JR).

The strong involvement of the Medicine and Family Medicine departments at UU and UMN relates to the academic appointments of the HELM leaders and has no bearing on the selection of HELM participants, who come from various disciplines.

At both institutions, HELM is supported by External Advisory Boards (EABs) with national experts in health equity, leadership, and mentoring. EABs for each respective program meet annually to review progress, provide feedback, and make recommendations for continued improvement.

## HELM Fellow demographics

Postdoctoral fellows (PhD or equivalent), residents and fellows in clinical training, and faculty at the assistant professor level or equivalent are eligible for HELM, which accepts 12–15 Fellows annually. The competitive application involves a statement of interest, brief vitae, and, at UU, an email of support from the prospective Fellow’s supervisor (e.g., chair). If all spots are filled, applicants are waitlisted and/or encouraged to reapply in the future. Fellows’ primary mentors are informed of their participation in HELM.

To date, the UMN program has completed 10 cohorts and supported 134 Fellows from 9 schools and colleges (Medicine, Public Health, Pharmacy, Liberal Arts, Social Work, Biological Sciences, Food, Agricultural and Natural Resource Sciences, Education and Human Development, Veterinary Medicine) across the University of Minnesota, including faculty from the Twin Cities, Duluth, and Rochester campuses; and VA Medical Center and Hennepin Healthcare in Minneapolis. At UU, the program has graduated 73 HELM Fellows in 6 cohorts from 4 schools and colleges in the health sciences (Medicine, Nursing, Dentistry, Pharmacy), Huntsman Cancer Institute, Nora Eccles Harrison Cardiovascular Research and Training Institute, and Eccles Health Sciences Library at the University of Utah, and the VA Medical Center in Salt Lake City.

Table 1 summarizes Fellows’ demographic data. At UMN, 73% were women, and 55% belonged to a URSM racial or ethnic minority group (including Middle East or North African and NIH-defined categories of Black or African American, Native American Indian or Alaska Native, Native Hawaiian or Other Pacific Islander, Hispanic or Latino, Asian or Asian American). At UU, 67% were women; 84% identified as belonging to a URSM racial or ethnic group. Table 1 also presents information about Fellows’ academic positions at the time they participated in HELM and their terminal degrees. At UMN, 57% were faculty, 31% were postdoctoral fellows, and 11% were residents or fellows in clinical training. At UU, 44% were faculty (including visiting faculty), 38% were postdoctoral fellows, and 10% were fellows in clinical training. At UU, more participants held PhDs (55%) than MDs or MD/PhDs (37%).

**Table 1.** Demographic data for Health Equity Leadership and Mentoring Fellows

	UMN (*N* = 134)	UU (*N* = 73)
Variable	*n* (%)	*n* (%)
**Race and ethnicity**		
Black or African American	29 (22)	18 (25)
American Indian or Alaska Native (AI/AN) or Native Hawaiian or Other Pacific Islander (NH/PI)	5 (4)	3 (4)
Hispanic or Latino	14 (10)	18 (25)
Asian or American Asian	15 (11)	15 (21)
Middle East or North African (MENA)	1 (1)	7 (10)
white	60 (45)	8 (11)
>1 race/ethnicity	10 (7)	0 (−)
prefer not to disclose	0 (−)	4 (5)
**Gender and gender identity**		
Female	98 (73)	49 (67)
Male	33 (25)	23 (32)
Other/prefer not to disclose	3 (2)	1 (1)
**Academic rank/title**		
Faculty: assistant professor	74 (56)	25 (34)
Faculty: associate professor	1 (1)	4 (5)
Faculty: visiting	0 (−)	4 (5)
Postdoc (PhD)	41 (31)	28 (38)
Resident/fellow (MD)	10 (7)	7 (10)
Research associate	5 (4)	2 (3)
Other	2 (1)	3 (4)
**Degree**		
MD		24 (33)
PhD		40 (55)
MD/PhD		3 (4)
Other		6 (8)

While we did not collect data on research areas or experience, Fellows’ departmental affiliations, the information they volunteered during the year, and their stated interest in health equity suggest that HELM Fellows have characteristics similar to populations participating in CTSA programs. For instance, the health equity focus is more applicable to translational and clinical research, and HELM has indeed seen only a few Fellows from basic science departments. HELM’s target audience is trainees and early-career faculty, which suggests further similarities between HELM Fellows and CTSA populations.

## Conceptual framework and HELM domains

**Figure 1.**
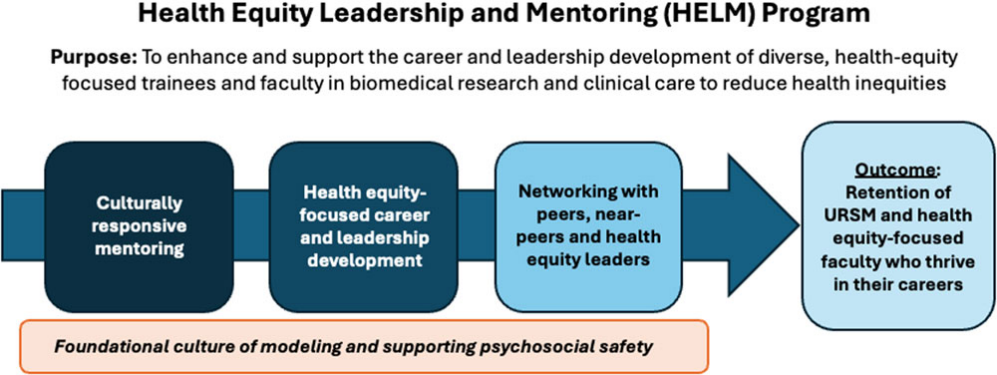
Health Equity Leadership and Mentoring conceptual framework.

Figure 1 shows the evolving conceptual framework of HELM. Through their prior experience mentoring URSM trainees and a desire to specifically address the unique challenges experienced by trainees and faculty who identify as URSM and/or have committed to careers focused on health equity, the founding directors of HELM purposefully aimed to create an environment that fosters support and enthusiasm for the career goals and milestones of HELM Fellows. The foundation for all programmatic activities is this intentional cultivation of a safe (or “brave”) space for Fellows. HELM directors and mentors model and, together with Fellows, contribute to a HELM culture in which people feel safe, lift each other up, create a sense of belonging, and practice speaking up. This culture of psychosocial safety is the hallmark of HELM and the trait that runs across the three domains of the program: culturally responsive mentoring by established faculty; career and leadership development of Fellows; and networking opportunities with peers, internal faculty across the career spectrum, and external health equity leaders.

### Mentoring

HELM matches Fellows with mid-career and senior faculty for mentoring in professional career planning and career development. HELM mentoring relationships do not replace or compete with traditional discipline-specific or research mentoring; instead, they provide a safe space that considers cultural identity – including race, ethnicity, nationality, gender/gender identity, and sexual orientation – as an important factor that shapes career goals, professional experiences, and recruitment, retention, and promotion outcomes.

The HELM program requires mentors who have deep experience with mentoring diverse faculty and trainees. Both institutions have a pool of 25–30 prospective mentors who are established faculty at the advanced associate or full professor level, including chairs and division chiefs. We recruit mentors each summer through email advertisement and confirm with existing mentors that they would like to continue their engagement with HELM. Research suggests that racial and ethnic mentor-mentee concordance positively impacts mentoring relationships for URSM trainees, yet the demand for mentors with diverse backgrounds exceeds their availability on most campuses [38]. This reality is reflected in the racial and ethnic composition of HELM mentors at UMN and UU, which are predominantly white institutions. Some HELM mentors have URSM backgrounds, but the majority are white, and women are overrepresented. HELM addresses the lack of demographic mentor-mentee concordance by seeking mentors with a demonstrated record of successful mentoring and equity leadership as evidenced by reputation, products, and administrative leadership roles.

HELM also provides mentors with mentoring resources, including the NIH-funded Culturally Aware Mentoring (CAM) curriculum. CAM is built on evidence that culturally responsive mentoring can be learned to effectively communicate and address cultural differences between mentor and mentee [10,17]. CAM training is delivered by CIMER-trained facilitators (at UU, these are members of the HELM leadership team, who became certified facilitators specifically to train HELM mentors). However, HELM does not require that mentors complete the CAM workshop. HELM mentors are a self-selected group of whom most have participated in leadership development opportunities specifically addressing health equity and workforce diversity. In our experience, mentors opt to stay in the pool for consecutive years; we have a minimal drop-out rate.

HELM matches Fellows and mentors in a monthlong process during which Fellows meet with several prospective mentors based on HELM directories that include detailed participant bios outlining interests and experiences. At UU, HELM hosts a Mentor Open House, a speed-networking event during which Fellows and prospective mentors engage in a series of short one-on-one conversations. The program prepares Fellows with conversation starters (e.g., open-ended guiding questions, elevator pitch). At UMN, the program schedules up to 4 individual meetings for each Fellow to identify their preferred mentor. After interviewing each other, HELM Fellows and mentors declare their match preference. The program completes a thorough review and matches Fellows and mentors according to their stated preferences, which do not necessarily meet research interests or clinical practice. Once mentors and Fellows are matched, they agree to meet at least three times during the program duration. Fellows schedule meetings and are responsible for the agenda.

HELM trains Fellows with excerpts from the NRMN-based Entering Mentoring curriculum. This prepares Fellows to use time with mentors productively as well as begin mentoring relationships with trainees of their own. HELM provides Fellows with an Individual Career Development Plan (IDP) template and instructions on how to use it to kick off the mentoring relationship with their mentor. Each Fellow-mentor dyad is paired with a HELM (co-)director, who supports the mentoring relationship and meets with Fellows individually three times per year to provide additional mentoring.

We designed HELM as a cohort experience to foster camaraderie among Fellows. As HELM evolved, we realized that interactions between Fellows extended beyond casual socializing to peer mentoring, manifest in Fellows’ exchange of career advice, expressions of support, celebrations of success, and occasional professional collaborations. Monthly HELM sessions intentionally include time for Fellows to connect as peer mentors.

### Health equity-focused career and leadership development

The career development domain is centered on HELM’s core curriculum of monthly two-hour sessions, which include workshops, book discussions, panel discussions, and presentations from internal and national speakers recognized for their health equity and workforce diversity scholarship and leadership. Generally, sessions do not focus on research or clinical work but on career and leadership development at the intersection of academic health professions and health equity research or clinical care. Selected topics include “A Black academic’s guide to getting tenure,” “Demystifying (and overcoming) imposter phenomenon,” “Racial battle fatigue,” “Mapping your developmental network,” “Developing a health equity framework,” “Preparing for social justice leadership positions,” and “Inclusive leadership and equity system change.”

Roughly one-third of the session content focuses on the development of leadership knowledge and skills. At UMN, the leadership development series “Equity Leadership Journey” (facilitated by AAW) uses a participatory approach that scales up the personal to the systemic, using personal practice, dialogue, facilitation, and the co-creation of innovation to address complex challenges. At UU, HELM leadership sessions focus on developing a path to leadership informed by awareness of one’s professional strengths combined with a personal philosophy of “Why” (e.g., Why health equity? Why academic medicine?) [39]. To date, HELM has not specifically measured the development or growth of leadership skills through pre- and post-intervention assessments; however, leadership discussions are repeatedly rated highly in post-session evaluations. Future plans include assessing leadership competencies at the beginning and end of the HELM experience to more fully understand the impact of the program in this domain.

We often invite members of our respective EABs to present, and we facilitate leadership panels that include URSM and/or health equity-focused faculty. Frequently, sessions feature the personal career journeys of our speakers. We have observed that Fellows are particularly interested in learning about leaders’ career challenges that were magnified by cultural differences. Due to the lack of URSM role models and opportunities to hear authentic personal experiences of successful faculty, these discussions have been highly insightful and validating to Fellows. By speaking up about challenges and inviting Fellows to ask direct questions, presenters shape the HELM culture to be a place of belonging, and they equip Fellows with practical knowledge for navigating academic medicine. Modeling health equity leadership this way further develops Fellows’ leadership knowledge and skills.

HELM sessions are held in person (UU) or as a hybrid of in-person and virtual seminars (UMN). As part of supporting the Fellows’ application into the program, department chairs commit to protect participants’ time to fully engage with monthly sessions.

Fellows receive career development funds through HELM (between $250 and $500 per Fellow, to be expended during the duration of the program), which they use to supplement relevant conference travel or other career development activities.

### Networking

Fellows have access to alumni of previous HELM cohorts, mentors, program directors, internal speakers, and EAB members. Fellows are encouraged to work with their mentors for additional introductions to health equity-focused and workforce diversity scholars and networks. To foster community building, each HELM session provides lunch for peer mentoring and networking. The last session is a HELM closing celebration to which mentors and internal presenters are invited. Previous cohorts have expressed interest in social gatherings outside the HELM curriculum. One UU HELM cohort created a social media channel to which alumni and new Fellows are invited, and HELM Fellows at both institutions report that they stay in touch and maintain their relationships as HELM alumni. These interactions are not monitored by the program.

**Figure 2.**
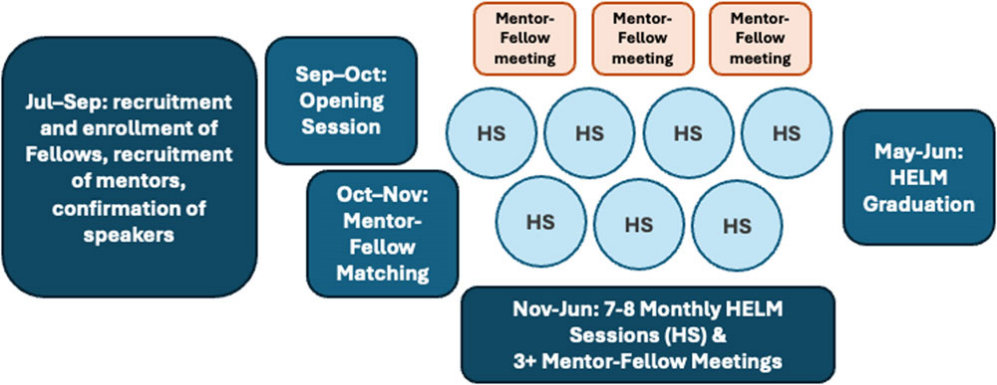
Health Equity Leadership and Mentoring program timeline.

Mentoring, career development, and networking activities coincide with the academic year (Figure 2).

## Evaluation

At the end of each HELM session, Fellows are asked to complete an anonymous survey. At UMN, the 8-item survey asks Fellows to indicate the extent to which they agree with statements such as “the quality of the seminar met my expectations,” “the topics discussed are relevant,” and “I learned something new that can be applied in my professional life.” At UU, the 5-item survey asks Fellows to rate the clarity, session objectives, educational value, level of appropriateness, and overall experience of sessions; in addition, Fellows are encouraged to provide comments in open-text field.

After the conclusion of HELM, Fellows respond to a questionnaire about the overall program. UMN uses a 4-item survey that asks Fellows to respond on a scale from 1 (= extremely unhelpful/unlikely) to 10 (= extremely helpful/likely). Evaluations have shown excellent satisfaction and appreciation for HELM. The mean score for each of the items over the duration of the program has been remarkably consistent, ranging from 7.9 to 9.8: “How would you rate the overall program?” (8.7); “How would you rate the overall format of the program?” (8.6); “How would you rate the overall experience with your mentor?” (8.6); “How likely are you to recommend the HELM program to others?” (9.1). UU uses similar questions regarding the overall program, experience with mentors, and likelihood of recommendation (see Table 2). HELM Fellows at UU ranked their experiences with senior mentors lower than the program overall, yet they still expressed high satisfaction.

**Table 2.** University of Utah Health Equity Leadership and Mentoring overall program evaluations 2021–22 and 2022–23 (*N* = 22)

	1	2	3	4	5
	*n* (%)	*n* (%)	*n* (%)	*n* (%)	*n* (%)
How do you rate the overall program? (from 1 = not helpful to 3 = neutral to 5 = extremely helpful)	0 (−)	0 (−)	0 (−)	3 (14)	19 (86)
How do you rate the overall experience with mentors? (from 1 = not helpful to 3 = neutral to 5 = extremely helpful)	0 (−)	1 (4)	2 (9)	5 (23)	14 (64)
How likely would you recommend this program? (from 1 = not likely to 3 = neutral to 5 = very likely)	0 (−)	0 (−)	0 (−)	4 (18)	18 (82)

Overall program evaluations also include open-ended questions to solicit feedback that might help contextualize the quantitative evaluation data. The approach is akin to that taken in course evaluations and not designed as a qualitative study necessitating full thematic analysis of responses. Fellows commonly reported that the HELM safe-space culture that threads through the three HELM domains was one of the most beneficial aspects of the program. Indeed, the salience of this experience is reflected in the fact that the quotes below were elicited in response to separate qualitative questions, including “what was most affirming or helpful about the HELM program,” “what was most surprising,” and “what else would you like to tell us about your experience of HELM”:

I appreciated that every speaker took the time to affirm that our sessions were a safe space for discussion and learning. (UMN)

I really appreciated how open and honest the other fellows were through this journey. I thought that was really helpful because so many of them had been in a similar place as I am and they helped to reaffirm my feelings, but also to help me talk through things and realize what I was feeling. (UMN)

I felt surprised by the number of people who shared my challenges with navigating academia. Often my experiences feel isolating, so it was so helpful to be reminded that others are often experiencing similar things. (UMN)

I loved it when facilitators really held us accountable and gave us space to ask questions and be vulnerable. (UMN)

Keep up the good work. U-HELM created a safe space for us to have necessary but difficult discussions. This is especially necessary in today’s environment. (UU)

Thank you so much for hosting this program. This is the first time I’ve felt I “belonged” in the academy. I felt seen as a whole person as a U-HELM trainee. I am incredibly grateful for the individual experiences that were shared with us from all of the faculty, invited speakers, and my fellow U-HELM members. I would absolutely recommend this program. I think this kind of content would be a total game changer for all new/junior faculty. (UU)

The sense of belonging documented here, which developed especially among peers, can increase retention among diverse faculty and may serve as an important recruitment tool [40] for trainees to apply for internal positions upon completion of their training. To date, over 85% of UMN HELM Fellows who were not in a training role at the time of their participation have remained at UMN. At UU, 93% of Fellows who participated in HELM as faculty remain in faculty positions, and 12% of those have been promoted since their participation in HELM. All visiting professors who participated in UU HELM have transitioned into assistant professor roles. Fourteen percent of trainees who participated in UU HELM are UU faculty now.

## Challenges

HELM was designed as an in-person program, pivoted to virtual with the onset of the pandemic in March 2020, and then followed a hybrid format, remaining remarkably strong. While the 2019–20 cohort had already developed into a cohesive group that comfortably switched to an online format, 2020–21 Fellows successfully built a robust sense of belonging despite their remote experience of HELM, demonstrating both the program’s adaptability and its value to participants. HELM has returned to an in-person delivery of content, with 2 enduring changes post-pandemic: (1) The switch to a virtual format demonstrated both ease and cost-efficiency for hosting speakers. Thus, videoconferencing technology remains an available option for external speakers and EAB members to present and meet with Fellows in one-on-one or small-group meetings. (2) To accommodate a gradual return to in-person events in 2021–22, we added outdoor events in support of virtually delivered sessions. These social gatherings proved successful for networking, and UU frequently holds the HELM graduation in an outdoor space that includes Fellows, their mentors, and internal speakers.

In 2024, legislation was passed in Utah (House Bill 261) that made diversity, equity, and inclusion offices and titles unlawful in state institutions. HB 261 also requires that diversity programs be open to all identities. This has been true for many decades, but the law has limited the number of pathway programs and changed how recruitment for these programs is implemented. UU HELM remains unaffected by this law, as there are no identity requirements for participation. In response to HB 261, UU HELM offers additional resources, comradery, and space to educate on legislation that affects workforce diversity and to discuss strategies to continue advancing health equity research, education, and care.

## Conclusion

HELM is a unique model for mentoring in that it provides cohort-based health equity-focused career support and establishes a sense of belonging among Fellows. Preliminary evidence demonstrates that HELM enhances health equity leadership knowledge and skills, but additional alumni data will need to be collected as validation of the program curriculum in developing leadership competencies.

The program’s focus on health equity attracts trainees and early-career faculty from a wide variety of identities, many of whom are URSM. Over the past 10 years, since the initial conception of HELM, demand for the program has grown, and it has proven to be an effective retention tool, with potential use for recruitment as well. Despite legislation challenging the validity of diversity programs, HELM will continue to be a positive force in the career advancement of URSM trainees and faculty and support retention efforts toward greater diversity in biomedical research and clinical care. Since HELM is designed and open to all early-career faculty and fellows, it does not conflict with the wide array of diversity, equity, and inclusion legislation impacting many US states [41]. Thus, HELM is a model that could be applied nationally to support the next generation of committed and highly qualified health equity researchers, educators, and clinicians.
